# Deep learning networks reflect cytoarchitectonic features used in brain mapping

**DOI:** 10.1038/s41598-020-78638-y

**Published:** 2020-12-16

**Authors:** Kai Kiwitz, Christian Schiffer, Hannah Spitzer, Timo Dickscheid, Katrin Amunts

**Affiliations:** 1 Cécile and Oskar Vogt Institute of Brain Research, Univ. Hospital Düsseldorf, Heinrich-Heine University, Düsseldorf, Germany; 2 Max Planck School of Cognition, Stephanstrasse 1a, Leipzig, Germany; 3 Institute of Neuroscience and Medicine (INM-1), Forschungszentrum Jülich, Jülich, Germany; 4 Institute of Computational Biology, Helmholtz Zentrum, München, Germany

**Keywords:** Imaging, Cells, Nervous system, Anatomy, Computational science

## Abstract

The distribution of neurons in the cortex (*cytoarchitecture*) differs between cortical areas and constitutes the basis for structural maps of the human brain. Deep learning approaches provide a promising alternative to overcome throughput limitations of currently used cytoarchitectonic mapping methods, but typically lack insight as to what extent they follow cytoarchitectonic principles. We therefore investigated in how far the internal structure of deep convolutional neural networks trained for cytoarchitectonic brain mapping reflect traditional cytoarchitectonic features, and compared them to features of the current grey level index (GLI) profile approach. The networks consisted of a 10-block deep convolutional architecture trained to segment the primary and secondary visual cortex. Filter activations of the networks served to analyse resemblances to traditional cytoarchitectonic features and comparisons to the GLI profile approach. Our analysis revealed resemblances to cellular, laminar- as well as cortical area related cytoarchitectonic features. The networks learned filter activations that reflect the distinct cytoarchitecture of the segmented cortical areas with special regard to their laminar organization and compared well to statistical criteria of the GLI profile approach. These results confirm an incorporation of relevant cytoarchitectonic features in the deep convolutional neural networks and mark them as a valid support for high-throughput cytoarchitectonic mapping workflows.

## Introduction

The human brain is not only target of the application of artificial neural networks (ANNs) to study its organization, it also represents a natural network of enormous complexity and power, which inspired their development. This has created a unique, bi-directional relationship throughout the last decades between research on brain organization and the application and development of ANNs^1–7^. Trying to understand the details of how modern ANNs internally operate is an ongoing endeavour and prerequisite to explain their results^8,9^, and led to the emerging research field of explainable AI. Due to the special relationship between brain organization and ANNs, such insights are of special interest when applying ANNs to study brain organization itself.

**Figure 1:**
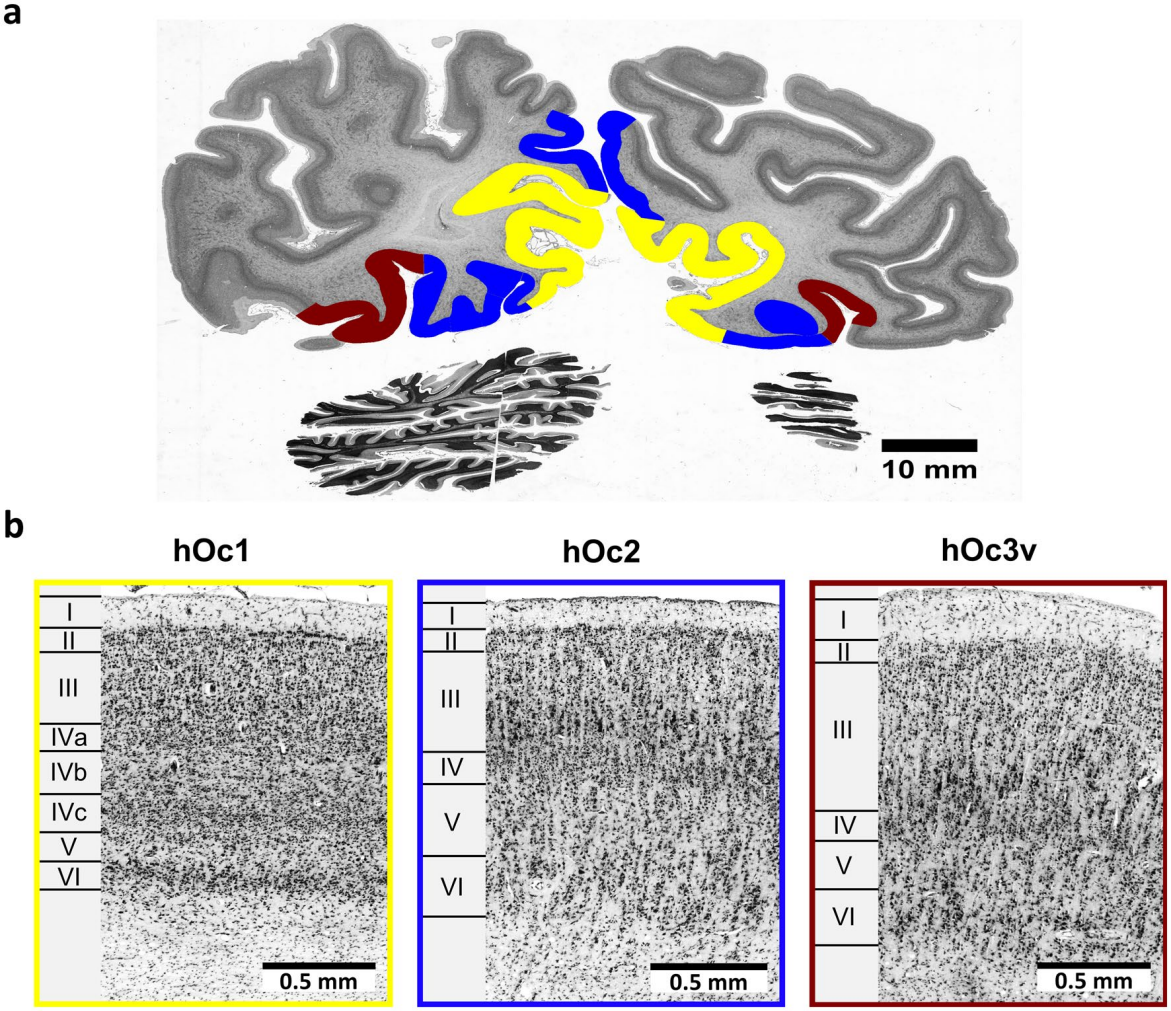
Cytoarchitecture of cortical areas of the visual system. (**a**) Histological cell-body stained section (section 901) from the occipital lobe of the BigBrain dataset with cortical areas hOc1 (yellow), hOc2 (blue) and hOc3v (brown)^16,17^. (**b**) Cytoarchitecture of cortical areas hOc1, hOc2 and hOc3v extracted from (**a**). Roman numerals indicate cortical layers. Area hOc1 is characterized by a prominent cortical layer IV, subdivided into sublayers IVa, IVb and IVc. Sublayer IVc shows the highest cell-density and constitutes the cortical input layer for visual information from the thalamus^53^. Cortical layer III and V contain small cells with the cell-sparse layer V being easily distinguishable from cortical layer VI^10,16^. In area hOc2, the size of pyramidal cells in cortical layer III steadily increases from upper to lower levels of the layer. Cortical layer IV is thinner than in area hOc1 and the contrast in density between cortical layer V and VI is not as high. The overall clarity of a columnar arrangement also appears increased^16^. Neighbouring area hOc3v has a moderate cell-density^16,17^. The three cortical areas show distinct structural–functional relationships revealed by neuroscientific investigations, including topographic organization^54–57^, columnar organization^58–60^ and interhemispheric connectivity^61–64^.

The brain contains neuronal networks formed by axons and dendrites, which connect neurons in different brain regions. Neurons of the cerebral cortex are organized in layers and columns^10^. The distribution, arrangement and presence of neurons (*cytoarchitecture*) differs between brain regions and is associated with connectivity and functional differences^11,12^. Cytoarchitecture can be studied in histological sections stained for cell bodies^13^. Traditional cytoarchitectonic features include cell size, cell density, laminar thickness and arrangement, columnar arrangement of cells, cellular clustering, cortical thickness, as well as the sharpness of the white matter/grey matter border^10,11,13–15^. Figure 1 illustrates the cytoarchitecture of the primary visual cortex (Brodmann Area 17, hOc1, or V1, from here on called hOc1), the secondary visual cortex (hOc2, Brodmann Area 18, or V2, from here on called hOc2), and the ventrally adjoining area hOc3v^16,17^, which are part of a complex biological network for processing visual information^18^. While all three areas show the typical 6-layer structure of the isocortex, they differ with respect to their cytoarchitecture and role in information processing.

Previous studies of our own group have analysed the cytoarchitecture of areas hOc1, hOc2^16^ and hOc3v^17^. Borders between the areas were identified based on computerized image analysis and statistical tests^16,17^. Such methods based on quantitative measures enable a reproducible identification of borders^19–22^. The current state-of-the-art method for quantitative cytoarchitectonic analysis is based on the grey level index (GLI) as a measure of the volume fraction of cell bodies extracted along traverses^19^. The latter are defined along the Laplacian field from the cortical layer I/layer II border to the white matter border on GLI images^20,21,23^. The resulting GLI profiles reflect the cytoarchitecture and feature vectors are extracted to analyse changes in cytoarchitecture while moving across the cortical ribbon. The feature vectors contain the mean GLI value and the first four central moments about the mean: mean, standard deviation, skewness and kurtosis, as well as values of the differential quotient of the profile^19,21^. A sliding-window approach captures borders between cortical areas based on multivariate difference functions of the feature vectors^19^. This approach has allowed to identify areas in serial histological sections, to 3D reconstruct their extent, and to compute probabilistic maps as part of the *Julich-Brain* atlas^11,24–26^. It has been applied for more than 100 areas.

Alternative approaches have been proposed for cortical mapping, e.g. excess mass functionals in the feature vectors to establish a relation to differences in cortical lamination in consecutive profiles^22^. This reduces the complex shape of the profile to the number of local peaks and their differences^22^. Others have applied wavelet analysis to bundle profile data in large wavelet coefficients^27^. However, it is not always straightforward to interpret such transformations of the extracted profiles with respect to the original histological data since it is necessary to determine which features of a wavelet transform should be analysed in a second step^22^. Additionally, wavelet analysis represents profile descriptions at an abstract level that can hardly be related to underlying cytoarchitectonic properties of the histological tissue.

All approaches have in common that the extracted profile features only partly reflect traditional cytoarchitectonic criteria, but focus on statistical image criteria to detect laminar differences in the cellular pattern. The feature vector for statistical analysis in the current GLI profile approach allows to interpret them with respect to cytoarchitecture, e.g., mean cell packing density (mean GLI feature), or differences in cell density between supra- and infragranular cortical layers (e.g., skewness feature)^21,23^. However, such a reasoning cannot be made unequivocally since one and the same GLI value can result from a lower numerical density of large neurons and a higher density of small neurons^23^. While cytoarchitectonic analyses in mapping studies have benefited significantly from the GLI profile approach, recent developments of high-resolution models like the BigBrain dataset with more than 7400 stained histological sections^28^ challenge the throughput for future studies.

Deep learning techniques constitute a new and promising alternative in the dynamically evolving field of medical image analysis^29–33^, which potentially enable the segmentation of cortical areas in more sections as compared to the GLI profile approach. Deep ANNs have already led to robust and accurate results for cell detection in histopathological images^29,34–36^. The U-Net architecture^34^ is highly effective for biomedical image segmentation in this regard by using a deep convolutional neural network (CNN) approach, which we adapted for segmenting cortical areas on histological data in our own lab. It showed that the approach generates spatially consistent segmentations across sections that are transferable to other brains with high throughput^37,38^.

To further evaluate whether this approach is adequate to support cytoarchitectonic brain mapping, an in-depth comparison between the current GLI profile approach and deep learning-based mapping is required. We therefore analysed the internal structure of deep CNNs trained to segment different cortical areas in images of cell-body stained histological sections of the human brain. This included to evaluate in how far the internal structure of the trained networks reflect traditional cytoarchitectonic features on the cellular and laminar level of cortical areas. In addition, the laminar and cellular features reflected by the current GLI profile approach were compared to the features learned by the networks, and correspondences and dissimilarities between mapping results were analysed in regions of interest. The study was performed in the visual cortex of the BigBrain dataset—a frequently used, high-resolution brain model, for which all sections were histologically processed, stained, imaged and 3D-reconstructed^28^.

## Methods

### Cytoarchitectonic mapping based on GLI profiles

Cytoarchitectonic analysis was performed on histological sections of the BigBrain dataset^28^. This dataset consists of 7404 coronal, 20 µm thick, cell-body stained sections of a complete paraffin-embedded human brain^28^. The brain was originally obtained in accordance to legal and ethical regulations and guidelines as part of the body donor program of the Department of Anatomy of the Heinrich Heine University Düsseldorf. The body donor (65 years old, male) gave written informed consent for the general use of post-mortem tissue for aims of research and education. All usage in this work is covered by a vote of the ethics committee of the Medical Faculty of the Heinrich Heine University Düsseldorf (#4863). The numbering of the dataset starts at the occipital pole (section 1) and ends at the frontal pole (section 7404). For cytoarchitectonic analysis, a region of interest covering the primary visual cortex (hOc1) with its distinct cytoarchitecture, and the surrounding secondary visual cortex (hOc2) was chosen (Fig. 1). Both areas reach from the occipital pole to the parieto-occipital sulcus^16^ and are located between sections 1 and 2461 in the BigBrain dataset. The 3D-reconstructed BigBrain dataset and annotations of the areas are available online (https://interactive-viewer.apps.hbp.eu/).

**Figure 2:**
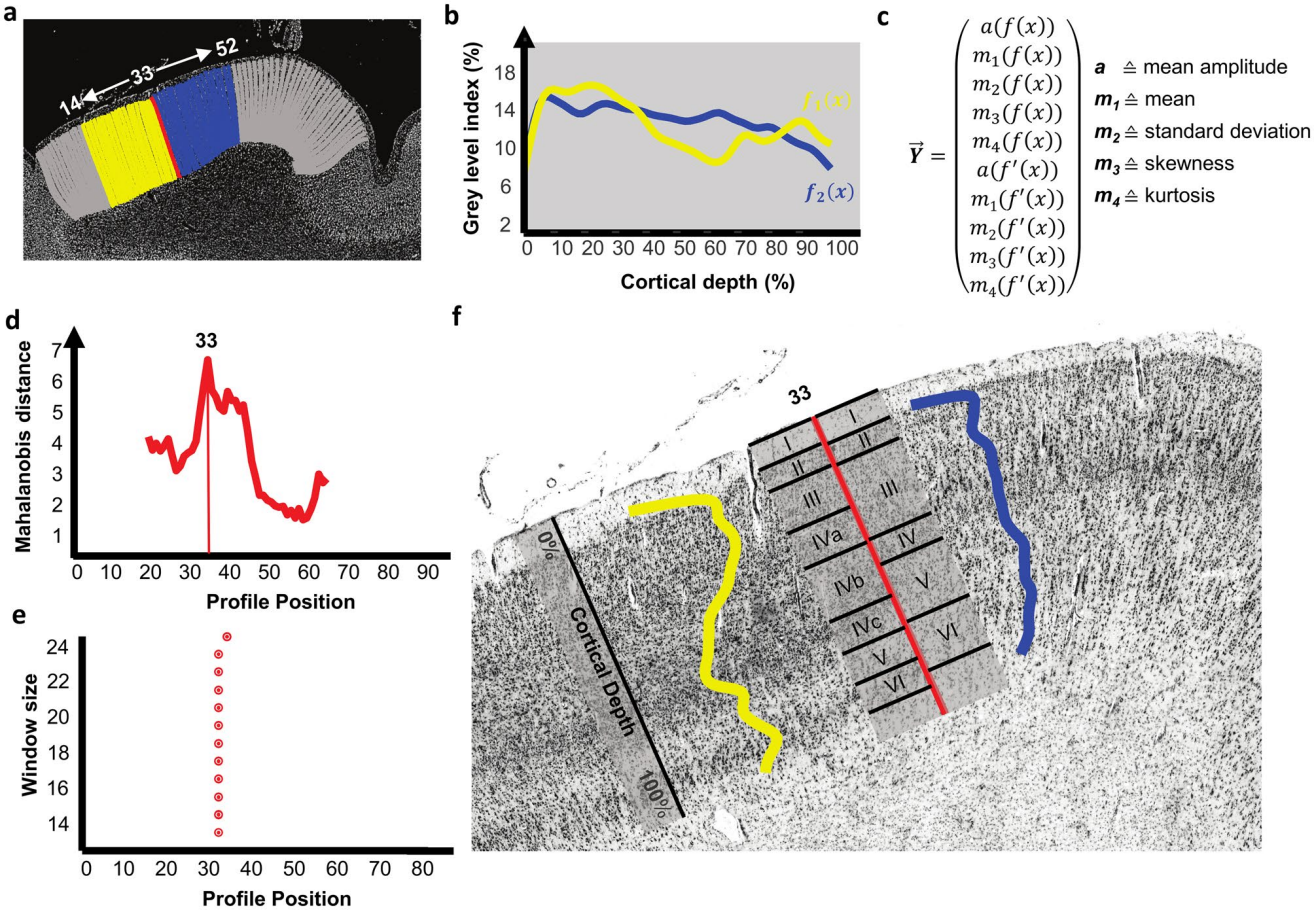
Illustration of the GLI profile approach for cytoarchitectonic analysis^19–21,23,47^. (**a**) shows profiles (19 yellow and 19 blue) around a target profile (red, number 33) on a GLI image of a region of interest in the occipital lobe of the BigBrain dataset^28^. The graph in (**b**) depicts mean profiles from each of the 19 profiles from (**a**). (**c**) shows the feature vector used to quantify the shape of the mean profiles in (**b**) by interpreting them as frequency distributions. The feature vector consists of the mean amplitude of the distribution, the first 4 central moments as well as the first derivatives of the later. (**d**) illustrates the multivariate Mahalanobis distance metric between feature vectors generated from a sliding window approach with window size 19. It indicates a significant global maximum of differences between feature vectors at profile position 33. Significant global maxima for different window sizes in (**e**) confirm the detection of a border at position 33. (**f**) shows the detected border (red bar) displayed on the histological section used in (**a**). Roman numerals indicate cortical layers. Superimpositions of the profiles from (**b**) reveal correspondences to cytoarchitectonic features and reflect differences in lamination and cellular composition between cortical area hOc1 on the left and hOc2 on the right side of the detected border^25^. These include a clear differentiation of cortical layer IV reflected by differences in GLI profile shapes, as well as differences in the excess of the profile shapes at cortical depths relating to cortical layer IVc to VI in area hOc1 and IV to VI in area hOc2. In area hOc1, the broad and cell-dense cortical layer IVc contrasts the cell-sparse layer V which in return can easily be distinguished from the darkly stained layer VI. Whereas in area hOc2 the thinner cortical layer IV does not stand out from the less distinguishable layer V and VI to the same amount in the GLI profile shapes.

The GLI profile approach was performed on three digitized sections (section 0961, 1021 and 1081) covering a distance of 2.4 mm. They represent the centre of the designated region of interest in the BigBrain dataset. Mean profile shapes have been extracted and borders between hOc1 and hOc2, as well as to ventrally and dorsally neighbouring areas hOc3v^17^ and hOc3d^39^ have been identified (Fig. 2). The resulting mappings on sections 0961 and 1081 served for training the deep CNNs. Mappings on section 1021 constituted a reference for analysing the CNNs’ internal structure and validating their segmentation performance.

### CNN based cytoarchitectonic mapping

**Figure 3:**
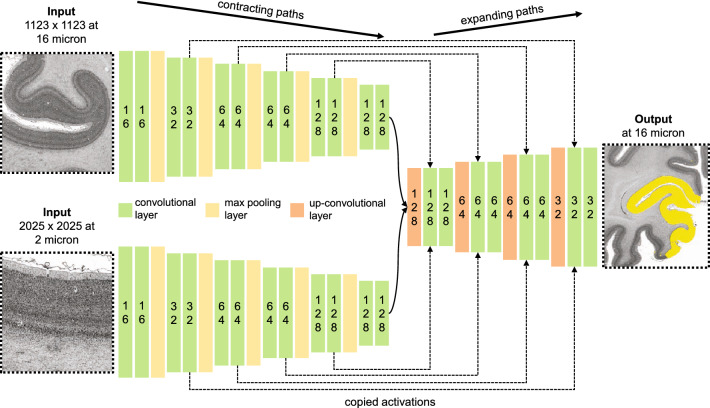
Scheme of the used CNN architecture inspired by the U-Net^34^. The blocks in the contracting branches consist of two convolutional layers and a pooling layer. The output of each convolution is parsed through a batch normalization and a rectified linear unit (ReLU). Each block in the expanding branch consists of an up-convolutional and two convolutional layers, each followed by a batch normalization and a ReLU. High resolution activations from the contracting path are combined with the outputs of the up-convolutional layers in the expanding branch. Numbers in boxes indicate the number of network units in a respective layer. All branches consist of 864 network units leading to a total of 2592 units per CNN.

Two CNNs were trained to segment cortical areas hOc1 and hOc2 on all 119 sections in between the training sections. The network architecture of the CNNs consisted of 10 blocks with 24 network layers modelled after the well-established U-Net architecture^34^, including modifications proposed which have been shown to work well for the task of cytoarchitectonic area segmentation^37,40^. We trained two separate instances of the same CNN architecture for cortical areas hOc1 and hOc2 by using the mappings on the training sections as well as classified volume information of the BigBrain dataset in its 2015 version (https://bigbrain.loris.ca/main.php?)^41^, including grey matter, white matter and background classifications. Other than conventional U-Nets, each instance comprised a high- and a low-resolution contracting branch with a larger field of view connected to a single expanding branch (Fig. 3), allowing the model to efficiently capture fine-grained cytoarchitectonic features, as well as coarse-grained morphological properties of the surrounding tissue. All branches consisted of 864 network units leading to a total of 2592 units per CNN. As the use of fine-tuned weights from a pre-trained network has shown to be beneficial in comparison to the use of random initialized weights^42^, we adopted weights from a successful auxiliary deep learning model developed in our lab, which has proven to boost segmentation performance among visual cortices^38^. In each training iteration the CNNs were shown patches sampled equally from white matter, background, the cortical area of interest (hOc1 or hOc2) and other cortex to assure a balanced training. The high-resolution contracting branches of the CNNs were shown a 4.05 × 4.05 mm patch (2025 × 2025 pixels at 2 micron per pixel) capturing fine-grained cytoarchitectonic features; the low-resolution contracting branches were shown a 17.97 × 17.97 mm patch (1123 × 1123 pixels at 16 micron per pixel) to capture coarse-grained morphological properties of the surrounding tissue^40^.

### Feature visualizations and feature identification

In response to being presented with the validation section, each unit of the two CNNs generated an activation (*filter activation*). For an analysis of the internal structure of the CNNs, these filter activations were calculated by using the Rectified Linear Unit (ReLU) outputs within the high-resolution contracting and expanding branches of the hOc1 and hOc2 CNNs. To reduce differences among output values, a normalization to an interval of [0.0, 1.0] was applied with the smallest value larger than 0 serving as the lower bound. Thus, each filter activation constitutes a whole image whose resolution is defined by the network layer is was calculated from. This resulted in 2592 filter activations for each of the hOc1 and hOc2 CNNs (5184 in total) on the validation section. Since the number of dimensions (118.383.390 pixels on the validation section) exceeded the number of data points (2592 filter activations) by a factor of over 10.000, dimensionality reducing methods like a principal component analysis are not suited to categorize similar components among the filter activations. Therefore, a three-step categorization workflow was applied to evaluate whether the internal structure of the CNNs reflect traditional cytoarchitectonic features: (i) identification of groups of similar filter activations across each CNN; (ii) identification of characteristic filter activations for each layer of a CNN and (iii) identification of cytoarchitectonically relevant features among the characteristic filter activations. In detail:

(i) Mutual Information served as a metric to identify similar filter activations for each CNN. We adopted the idea from medial image registration techniques that make use of mutual information of images^43,44^. In our case, the normalized activation interval [0.0, 1.0] of the ReLUs of two filter activations were used and transformed into one-dimensional and two-dimensional histograms with a binning frequency of 255 to calculate the mutual information of two filter activations. The joint histogram was determined using

$${p}_{ij}^{xy}=\frac{1}{N}\sum_{x \in X}\sum_{y \in Y}1 \left(\frac{i}{256}\le x<\frac{i+1}{256}\right)\mathbb{*}1\left(\frac{i}{256}\le y<\frac{i+1}{256}\right)$$

relative to the size of the filter activation maps ($$N=\left|X\right|=\left|Y\right|)$$ with an indicator function ($$1)$$. This step was repeated for every combination of the 2592 filter activations of each CNN.

(ii) To identify characteristic filter activations for each layer of a CNN, a pairwise mutual information matrix for all filter activations was generated for each CNN. Compilations of twelve filter activations with the highest pairwise mutual information for each filter activation of a CNN served to identify characteristic filter activations.

**Figure 4:**
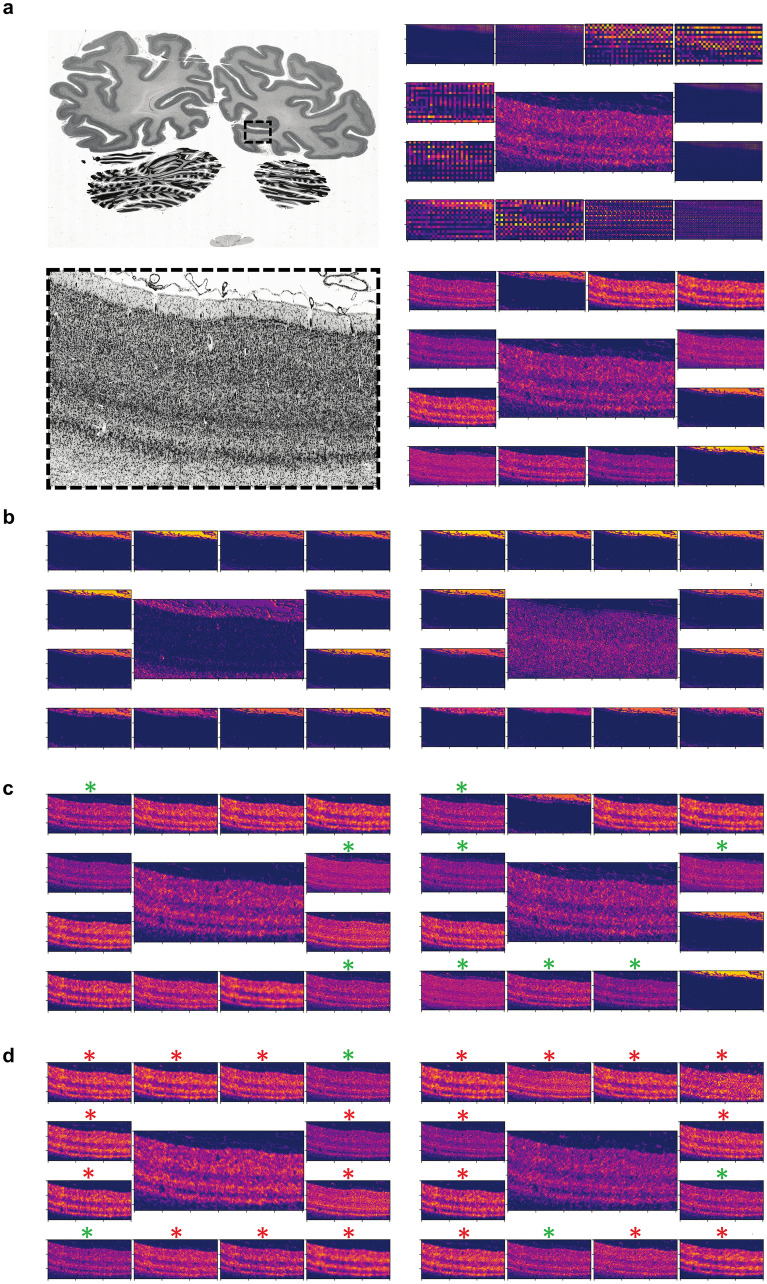
Mutual information compilations (**a**) and identification of characteristic filter activations (**b**–**d**) of network layer four from the hOc1 CNN. The two left images in (**a**) show the region of interest on the validation section (section 1021) in which the filter activations are visualized. Images on the right show two compilations of filter activations containing the lowest (upper right) and highest (bottom right) mutual information compared to reference filter activations (central images) across all network layers. (**b**–**d**) illustrate compilations of highest mutual information compared to different reference filter activations (central images). (**b**) contains two compilations with no characteristic filter activation in the middle since the criterion for similar appearance is not met. (**c**) contains two compilations with characteristic filter activations with all criteria met. Green asterisks mark similar-appearing filter activations. (**d**) contains compilations that appear similar to the reference but do not meet the criterion of at least three filter activations from the same network layer (green asterisks). Most similar-appearing filter activations belong to other network layers (red asterisks).

The compilations were analysed at a location within the validation section belonging to cortical area hOc1 or hOc2. Due to the increasingly lower resolution of the filter activations in deeper network layers (> layer 12) the whole section was analysed for the respective network layers. Filter activations were colour-coded for the analysis with a colour map that emphasizes lightness changes over changes in hue–a principle adopted from human colour perception^45^. As expected, filter activations with high mutual information exhibit very similar characteristics, justifying the choice of mutual information as a similarity metric (Fig. 4a). When a filter activation appeared similar to at least three other filter activations of the same network layer, it was determined to be characteristic for that network layer (Fig. 4b–d). This threshold was set to account for the different numbers of network units per network layer. A higher threshold prevents finding characteristic filter activations on superficial network layers with a small number of network units; whereas a lower threshold leads to a very high number of characteristic filter activations in deeper network layers with more units.

(iii) In a final step, the cytoarchitectonically relevant features among the characteristic filter activations were identified. Therefore, three categories of cytoarchitectonic features in accordance to traditional cytoarchitectonic features^10,14^ were defined:

- *first level features*, which are related to different shapes of cell bodies
- *second level features*, which are related to differences in thickness and composition of cortical layers
- *third level features*, which are related to differences at the level of cortical areas including their borders and extent.

When a characteristic filter activation fit into one of the three categories, it was identified to be cytoarchitectonically relevant and labelled a first, second or third level filter activation. The identification was performed by a neuroanatomical expert, who compared the characteristic filter activation to the three categories of traditional cytoarchitectonic features. Superimpositions of characteristic filter activations on the validation section enabled the identification of cytoarchitectonically relevant features among them. The size of the validation section alone constituted 15.1 Gigabyte with pixel dimensions of 94,321 × 80,326 (8-bit greyscale). To enable an analysis of such large datasets, we used the MicroDraw software^46^, due to its capability of displaying the superimpositions on large image data.

### Comparison of feature visualizations to the GLI profile approach

First, second and third level filter activations were compared to cytoarchitectonic features as revealed by the GLI profile approach^19,20,23^. The analysis included comparisons to cellular and laminar features reflected by the GLI profile approach, as well as mapping results of the border detection. The former was achieved by comparing first level filter activations to cell-related structures in a GLI image of the validation section. A comparison of second level filter activations to mean GLI profile shapes was used for a comparison of laminar features. Profile shapes were calculated from 25 profiles of the GLI image of the validation section. Locations of borders on the validation section detected by the GLI profile approach constituted a reference for comparing third level filter activations.

## Results

The analysis of the internal structure revealed a similar distribution of cytoarchitectonically relevant features among both CNNs trained to segment cortical areas hOc1 and hOc2. We detected first, second and third level filter activations in the hOc1 and hOc2 CNNs. First level filter activations were found on superficial network layers in the hOc1 and hOc2 CNNs, followed by second level filter activations on intermediate and third level filter activations in deeper network layers (Table 1). Thus, filter activations appeared in a similar successive manner within both CNNs. The most striking difference between the hOc1 and hOc2 CNNs constituted the internetwork quality of second level filter activations, which is described in more detail in the following sections.

**Table 1:** Distribution of first, second and third level filter activations among the network layers of the two CNNs.

	Distribution of first, second and third level filter activations in both networks					
	First level filter activations		Second level filter activations		Third level filter activations	
	hOc1 CNN	hOc2 CNN	hOc1 CNN	hOc2 CNN	hOc1 CNN	hOc2 CNN
Layer 1	x	x				
Layer 2	x	x				
Layer 3	x	x				
Layer 4	x	x				
Layer 5	x	x	x	x		
Layer 6	x	x	x			
Layer 7			x	x		
Layer 8			x	x		
Layer 9			x	x		
Layer 10			x	x		
Layer 11						
Layer 12						
Layer 13					x	x
Layer 14					x	x
Layer 15					x	
Layer 16					x	x
Layer 17					x	x
Layer 18					x	x
Layer 19					x	x
Layer 20					x	x
Layer 21					x	x
Layer 22					x	x
Layer 23					x	x
Layer 24					x	x

The requirements for a filter activation to be classified as a first, second or third level filter activation are described in the methods section. Crossmarks indicate the existence of first, second or third level filter activations among the characteristic filter activations of a specific network layer.

### Cytoarchitectonic features of cortical areas on the cellular and laminar level

**Figure 5:**
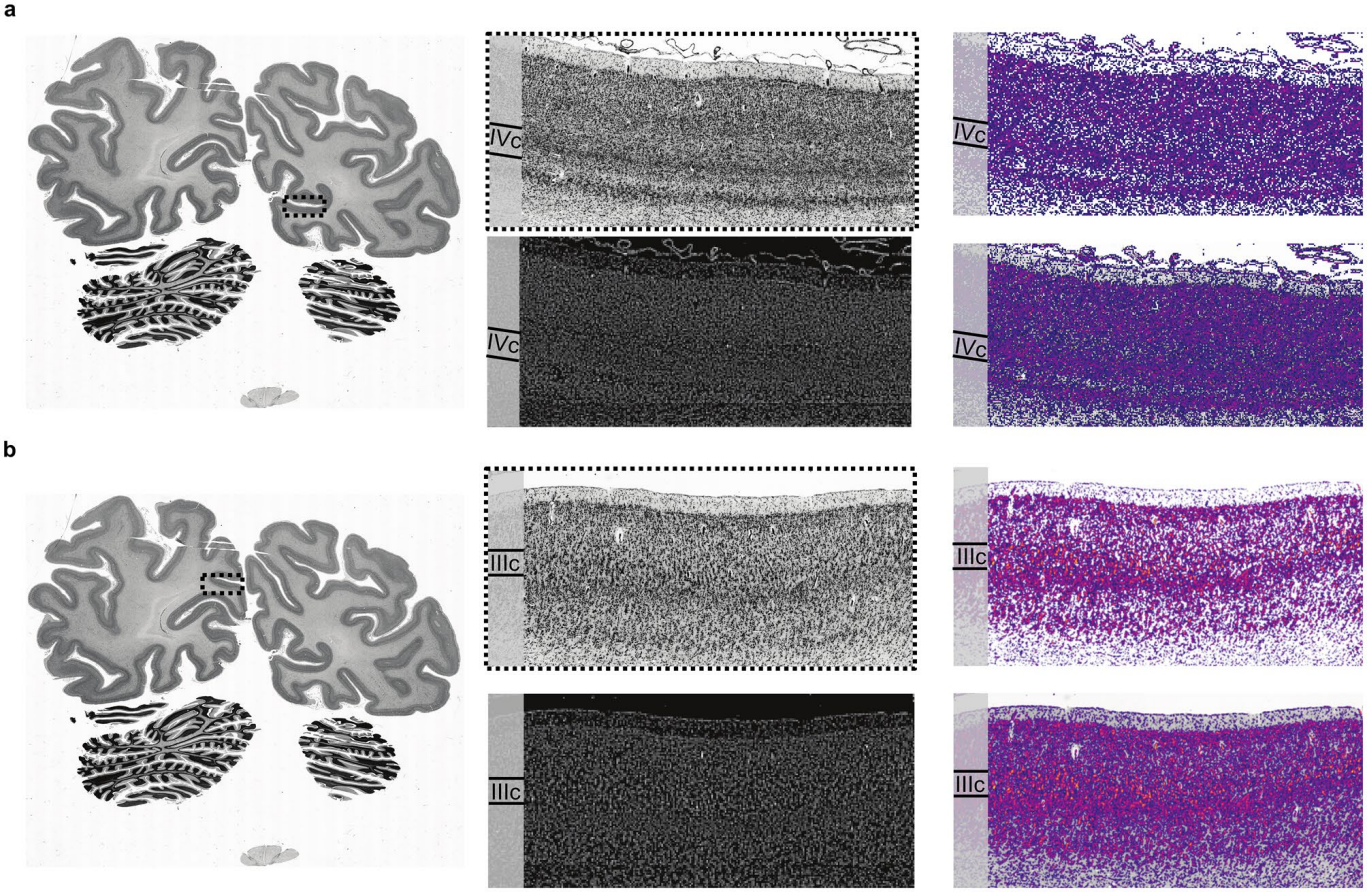
First level filter activations from network layer four of the hOc1 (**a**) and network layer one of the hOc2 CNN (**b**). The images on the left show the region of interest on the validation section (section 1021). The four images in the middle and right show microscopically magnified regions of interest of cortical area hOc1 (**a**) and hOc2 (**b**) depicted by dashed lines on the section on the left. In the middle, magnified histological cell-body stained images of the regions are displayed over the corresponding GLI images. Images on the right show the magnified characteristic filter activations (upper images) superimposed on cell-body stained images (lower images). Cortical sublayers with notable first level cytoarchitectonic features that are visible in the filter activations and the GLI images are marked by roman numerals.

First level filter activations were found on network layers one to six in the contracting branches of the hOc1 and hOc2 CNNs. In total, we found ten of them in the hOc1 CNN and 19 in the hOc2 CNN. They mainly responded to cell bodies in the cortex. When comparing the first level filter activations to the histologically stained validation section (Fig. 5a), cell-related properties of the histological image appear reflected in the filter activations. Examples for such correspondences are the cell-dense cortical layer IVc of area hOc1 (Fig. 5a) and large pyramidal cells in cortical layer IIIc of area hOc2 (Fig. 5b). In general, the first level filter activations did not show consistent variations.

**Figure 6:**
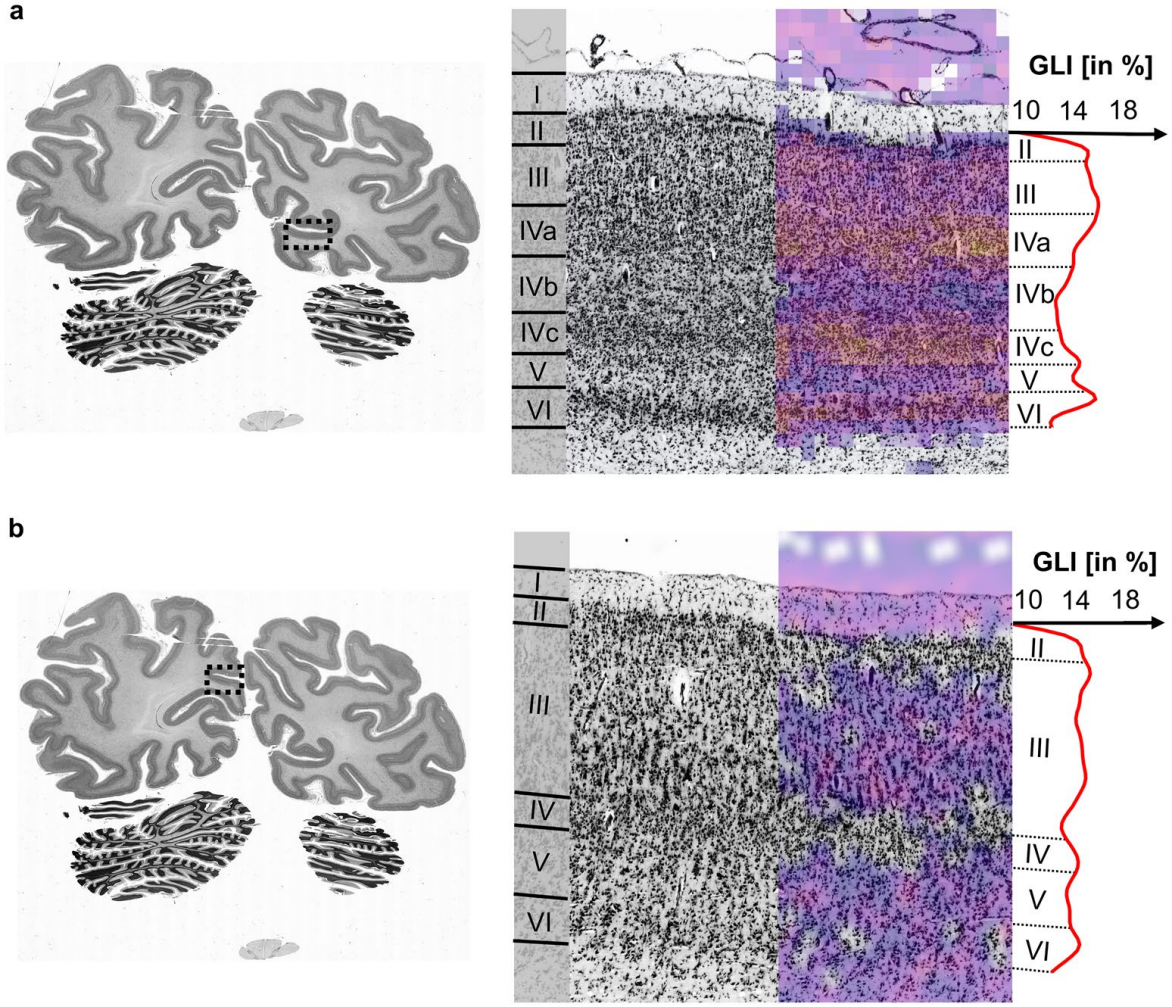
Second level filter activations from network layer 5 of the hOc1 (**a**) and network layer eight of the hOc2 (**b**) CNN**.** Big images on the right show the microscopically magnified regions of interest for cortical area hOc1 (**a**) and hOc2 (**b**) depicted by boxes on the left images (section 1021). They display cell-body stained histological images with the corresponding GLI profiles generated at this position. Roman numerals I to VI indicate cortical layers. Arabic letters indicate cortical sublayers. Superimposed characteristic filter activations show specific activations to cell-dense cortical layers of cortical area hOc1 (**a**) and cell-sparse cortical layers of cortical area hOc2 (**b**).

Second level filter activations occurred on network layers five to ten in the contracting branch of the hOc1 CNN, as well as five, seven, eight, nine and ten of the hOc2 CNN. In total, we found 45 in the hOc1 and 17 in the hOc2 CNN. A superimposition of a second level filter activation from the hOc1 CNN on histological data shows locally restricted activations within bounds of cell-densely packed cortical layers III, IVa, IVc and VI (Fig. 6a). In general, the 45 filter activations revealed only little variations in the strength of activations in cortical layer III. Cell-dense cortical layers IVa, IVc and VI showed consistently high activations. No second level filter activations responding to cell-sparse layers of the cortex were found in the hOc1 CNN.

Two different classes of second level filter activations occurred in the hOc2 CNN. The two classes constituted 17 second level filter activations showing activations to cell-dense cortical layers II and IV and four responding to the more cell-sparse cortical layers III and V. Figure 6b presents a superimposition of a second level filter activation from the hOc2 CNN which stays within bounds of cell-sparse cortical layers III and V. In general, the second level filter activations of the hOc2 CNN showed varying strengths of activations related to cortical layer I, the background as well as the white matter.

### Comparisons to cellular and laminar features reflected by the current GLI profile approach

The first level filter activations of the hOc1 and hOc2 CNNs revealed correspondences to cell-related structures in GLI images of the validation section. Examples constitute the dense cortical layer IVc in area hOc1 which appears as a dense white band in the GLI image (Fig. 5a) and big pyramidal cells in area hOc2 reflected by grossly-grained white dots in the GLI image (Fig. 5b). Note, that Fig. 5b depicts a part of cortical area hOc2 where the presence of big pyramidal cells in cortical layer IIIc alternates (left side: present; right side: non-present). This special cytoarchitectonic feature of cortical area hOc2^16^ is also reflected in the displayed filter activation and the GLI image.

Additionally, second level filter activations revealed correspondences to GLI profile shapes. Local maxima of the profile shapes reflect second level filter activations that respond to cell-densely packed cortical layers IVc and VI in area hOc1 (Fig. 6a). On the contrary, minima of the GLI profile shape correspond to the low filter activations of cell-sparse cortical layers IVa and V (Fig. 6a). The overall high respondence to cortical layer III also compares to the overall high GLI values in this layer. In area hOc2, local minima of the GLI profile shapes reflect second level filter activations responding to cell-sparse cortical layers III and V (Fig. 6b). High GLI values and local maxima of the profile shapes correspond to low filter activations in cell-dense cortical layers II and IV as well as the darkly stained cortical layer VI (Fig. 6b).

### Comparisons of deep learning based mappings to the GLI profile approach

**Figure 7:**
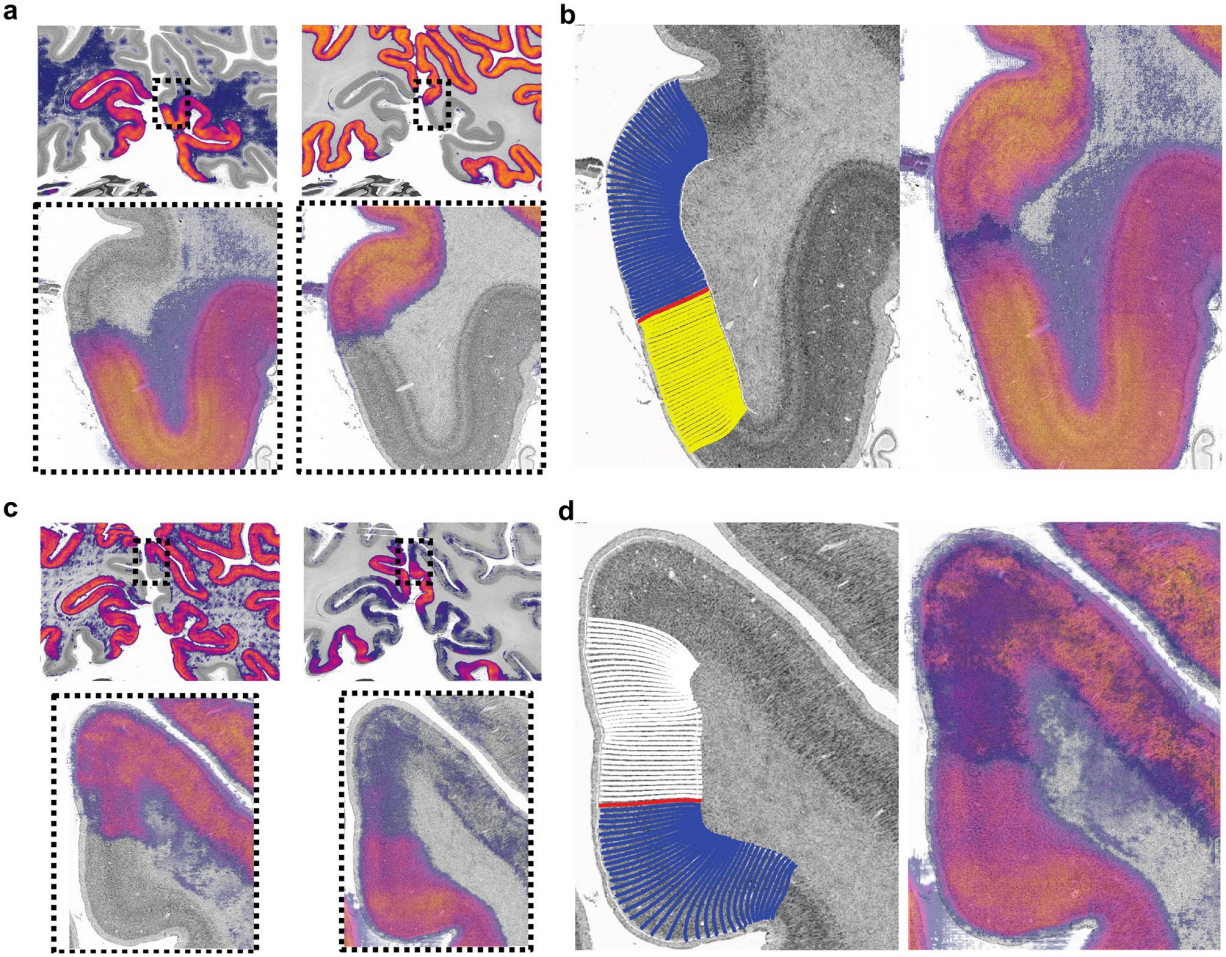
Third level filter activations of the hOc1 (**a**,**b**) and hOc2 (**c**,**d**) CNN. Images in (**a**) and (**c**) show two contrasting third level filter activations from network layer 23 of the hOc1 (**a**) and hOc2 (**c**) CNNs on the validation section (section 1021). The images in (**b**) and (**d**) compare the border detection result of the GLI profile approach (left) with the combined overlays of the two contrasting third level filter activations (right).

Third level filter activations are related to cortical areas and occurred in deeper layers 13 to 24 in the hOc1 and hOc2 CNNs (except for network layer 15 of the hOc2 CNN). In the hOc1 CNN, 123 of the third level filter activations showed clear cut activations labelling the extent of area hOc1, while ten filter activations showed consistent activations to the cortex surrounding area hOc1. The hOc2 CNN contained 41 third level filter activations labelling the extent of area hOc2, while 7 activations marked the surrounding cortex. When comparing the third level filter activations to the validation section, resemblances to the border position defined by the GLI profile approach become visible (Fig. 7). The superimpositions of two contrasting third level filter activations of the hOc1 and hOc2 CNNs are shown in Fig. 7a,c together with magnified images of the filter activations’ outer boundaries. Combined superimpositions of the filter activations show resemblances to the border positions defined by the GLI profile approach. In general, third level filter activations appeared more clear-cut in the hOc1 CNN than in the hOc2 CNN.

### Prediction precision

**Figure 8:**
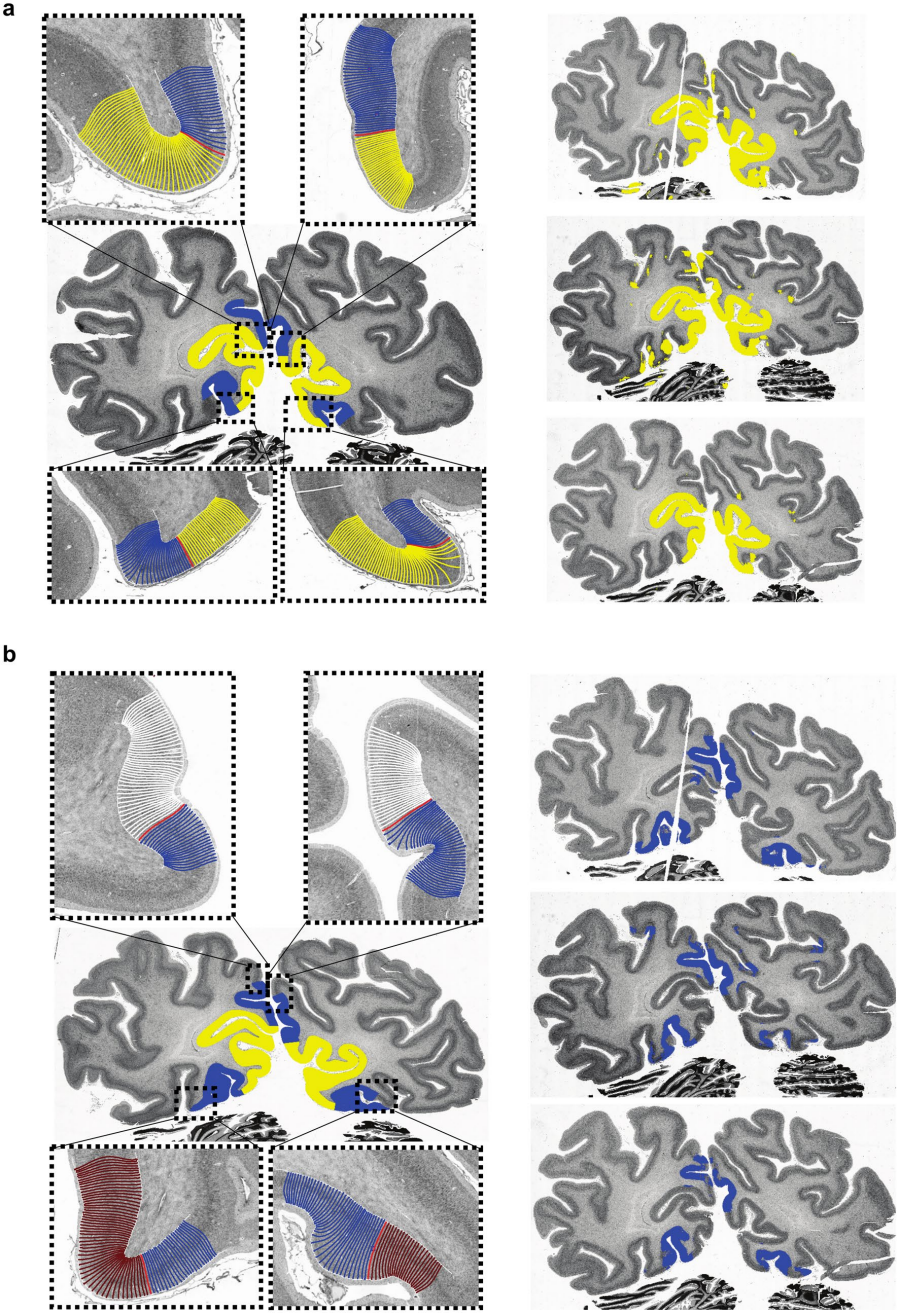
Segmentations of the hOc1 (**a**) and hOc2 (**b**) CNNs on three sections of the BigBrain dataset in comparison to borders detected by the GLI profile approach. Enlarged images of the training section (section 1081) in (**a**) show borders between cortical areas hOc1 (yellow) and hOc2 (blue). Enlarged images of the training section (section 0961) in (**b**) show borders between cortical area hOc2 (blue), hOc3d (white) and hOc3v (brown). Borders were detected using the GLI profile approach. The segmentations on three of the 119 sections (section 0991, 1036, 1066 from top to bottom) in-between the training sections are shown on the right in (**a**) for cortical area hOc1 (yellow) and in (**b**) for cortical area hOc2 (blue) for a comparison.

The two CNNs recognized areas hOc1 and hOc2 on all 119 unseen sections in-between the two training sections of the BigBrain dataset^28^. The predictions were anatomically plausible with regards to topography and neighbouring cortical areas^16^. Figure 8 shows that the pixel-wise predictions for cortical area hOc1 and hOc2 resemble the reference labels on the training sections. Single patches of falsely predicted pixels occurred, but were not connected to the accumulation of correctly predicted pixels that reflect cortical areas hOc1 and hOc2.

## Discussion

Cytoarchitectonic brain mapping has repeatedly profited from milestone achievements in computerized image analysis in the last 30 years^19–21,23,47^. Deep learning-based approaches have the potential to build workflows with a higher degree of automatization and hence increase the limited throughput of these techniques while at the same time providing independent verification of mapping results. Yet, profound insights in how far such techniques operate on criteria that resemble cytoarchitectonic features used by human experts are still lacking. This motivated the present study of deep CNNs, in which we investigated the learned network features for reflected cytoarchitectonic features and compared the internal network structure to statistical image properties used by the current GLI profile approach.

Our analysis confirmed that cytoarchitectonic features are indeed reflected in the internal structure of the deep CNNs and characterize different levels of organization, from cells to their arrangement in cortical layers, and entire cortical areas. Interestingly, the networks seemed to be more sensitive to capture the horizontal cortical organization in layers and sublayers, while the vertical arrangement in columnar structures did not seem to play a role. They inherently incorporate the interdependencies among the three types of features of cortical cytoarchitecture by representing their increasing complexity in an orderly manner from more superficial to deeper network layers. This is most likely due to the hierarchical structure of the convolutional network architecture, reflecting more and more complex features in deeper network layers.

A closer look on these representations revealed the existence of different subsets of filter activations depending on the cortical area. The hOc1 CNN for example contained only one set of filter activations that responded to cell-dense cortical layers on intermediate network layers. Area hOc1 receives massive input from the lateral geniculate nucleus of the thalamus, resulting in a very broad and cell-dense sublayer IVc^10^. In contrast layer V is cell-sparse (Fig. 1). These unique cytoarchitectonic features may have led the hOc1 CNN to develop a single set of filter activations that resembles cell-dense cortical layers. Similarly, the hOc2 CNN contains a feature set for cell-dense cortical layers as well. This is not surprising, since a prominent cortical layer IV (although not subdivided into sublayers) constitutes an important cytoarchitectonic feature of area hOc2 as well. Furthermore, layer IV helps to distinguish area hOc2 from area hOc3v which follows ventrally^16,17^. On the other hand, the hOc2 CNN contains one additional set of filter activations resembling cell-sparse cortical layers on intermediate network layers. The development of such a second set for cell-sparse cortical layers III and V stresses the reflection of cytoarchitectonic features in the hOc2 CNN in two ways alike. First, the clarity of the columnar patterns increases between cortical area hOc1 and hOc2^16^ and represents a cytoarchitectonic feature for differentiating the adjacent ventrally and dorsally located areas^17,39^. Since the columnar arrangement can mainly be observed in cortical layers III, V and VI^10^, a feature set for the cell-sparse layers III and V may help to incorporate this information. Secondly, the need for a second set is also underlined by the marked cell sparseness of cortical layer V compared to the ventrally adjoining area and the lack of big pyramidal cells in cortical layer V compared to the dorsally adjoining area. These adjacent areas also show a diminished increase in cell-size in cortical layer III^17^ and a lower cell-density in the upper part of cortical layer III^39^. In general, these observations support the notion that the CNNs are capable to capture distinct cytoarchitectonic features of cortical areas. They seem to be able to develop distinct representations of traditional cytoarchitectonic features on the cellular and laminar level of cortical areas.

In addition to the representations of traditional cytoarchitectonic features, the deep CNNs revealed correspondences to statistical image properties of the GLI profile approach. These include detailed correspondences between filter activations and GLI profile shapes, which constitute the essential measurement of the GLI profile approach. In addition, filter activations of the deep CNNs also correspond to cell-related features, whereas the GLI profile approach mostly focuses on laminar differences in the cellular pattern. This potentially enables the CNNs to encompass information about the columnar arrangement of cell bodies which constitutes an important cytoarchitectonic feature^10^. Filter activations from deeper network layers even reveal the possibility for the CNNs to have access to border positions as defined by the GLI profile approach (Fig. 7). These comparisons reveal that the learned internal feature representations of the CNNs compare well to the descriptive GLI profile shapes as well as to statistically defined borders of the GLI profile approach. Such correspondences to a well-established method provide further evidence for the CNNs’ potential in cytoarchitectonic brain mapping approaches.

While the two approaches show many correspondences, they do not share the same data basis. The deep CNNs operate directly on image patches extracted from cell-body stained sections. The GLI profile approach on the other hand, operates on GLI images, which estimate the volume fraction of cell bodies in small measuring fields of 20 × 20 microns by thresholding the original image intensities and summarizing foreground pixels in each field resulting in a lower dimensional image. For this reason, both approaches are likely to deal with locally restricted changes of cytoarchitecture differently. Here, such locally restricted cytoarchitectonic phenomena can be found in transition regions at the border of cortical area hOc1 and hOc2. These transitions regions have previously been described in myeloarchitectonic works as *border tuft* (“i.e. Grenzbüschel”) and *fringe area* “i.e. “Randsaum”)^16,48,49^. Right at the beginning of area hOc2, close to the border, the *border tuft* region hosts a distinct set of large pyramidal cells^16,49^ in layer III, accompanied by a very cell-sparse cortical layer V^16,49^. On the other side of the border, in area hOc1, the *fringe area* is cytoarchitectonically characterized by increased cell densities in cortical layers IVb, V and VI^16,49^. Additionally, cell sizes in cortical layer III of area hOc2 alternate^10,16,49^. These complex changes at a cytoarchitectonic border^11^ may explain the slight shifts of the assumed internal border representations within the CNNs (Fig. 7). Slight activations to the background and white matter in some of the filter activations may originate from different parameter sensitivities of the CNNs in comparison to the GLI profile approach. A comparison of both approaches should be considered with care therefore, although the deep CNNs seemingly incorporate traditional cytoarchitectonic features.

A deep understanding of internal network structure is mandatory to accept deep learning-based brain mapping as a valid support in future cytoarchitectonic mapping approaches. The present study provides first arguments for introducing deep learning-based brain mapping on a routine basis. First of all, it enables a direct assessment of incorporated cytoarchitectonic features via filter activation analysis. This constitutes an advantage in comparison to the feature vector of the GLI profile approach. Secondly, the analysis of the internal structure also revealed different spatial resolutions from cell-related to cortical layer-related to area-related features. This is especially important to capture the multi-scale organization of the cortex^11^—a circumstance that the current GLI profile approach does not mimic. It captures different spatial resolutions only indirectly in the direction of cortical columns by incorporating central moments like the mean and skewness in a profile shape’s feature vector.

A disadvantage is that the segmentation performance cannot be explained by the reflected cytoarchitectonic features per se. In fact, although highly improbable, the internal features that we identified to resemble cytoarchitectonic principles might not contribute to the final segmentation result at all. However, the resemblances of cytoarchitectonic principles is not a necessary nor a sufficient condition for successfully segmenting cortical areas. Several studies have been published in the past that were based on mathematical descriptions that were rather abstract, or tuned to detect architectonic gradients rather than to characterize the architecture itself. This resulted in reproducible and testable descriptions of borders without resembling traditional architectonic features directly^22,27,50^. The here applied CNNs are interesting in so far, as they reproduce what experts see to a certain degree, which introduces another level of confidence. It is possible that the CNNs may have developed non-intuitive features representing other (yet) unknown aspects of cortical cytoarchitecture. Such relationships have to be systematically studied in more detail in the future. Future advances in explainable image segmentation networks might allow us to assess the relevance of individual features for the actual segmentation outputs in a more reliable fashion. Additionally, analyses of more cortical regions with distinct cytoarchitectonic features would help to solve the question in how far the features detected by the hOc1 and hOc2 CNNs can be generalized to other areas, e.g., motor and higher association areas, or allocortical areas which contain a different number of cortical layers. This would go beyond the scope of this work, and remains a project of future research.

However, the amount of filter activations reflecting cytoarchitectonic features and the existence of different subsets suggests that deep learning with convolutional networks is able to capture cytoarchitectonic features. This is especially the case for cortical layer information. Such information is worth considering for future improvements of the deep learning approach. One possible option in this case is the explicit inclusion of information about laminar surfaces itself, which have recently been published for the BigBrain dataset^51,52^. Other incorporations of prior information, such as feeding in projected probabilistic maps^37^ or pre-training with an auxiliary task^38^ have already shown to improve the performance. Following this line, the present analysis gives valuable insights for such future considerations and provides strong evidence that deep convolutional networks are valid and suitable tools for high-throughput mapping workflows.

## Data Availability

The datasets generated and analysed during the current study are available in the EBRAINS repository [https://kg.ebrains.eu/search/instances/Dataset/78801754-16c1-4df2-9b2e-1b10c28a10c2].
